# sCD40L Is Increased and Associated with the Risk of Gestational Diabetes Mellitus in Pregnant Women with Isolated TPOAb Positivity

**DOI:** 10.1155/2022/2946891

**Published:** 2022-08-13

**Authors:** Xinxin Chen, Qingyao Wang, Xiangguo Cong, Shuyi Jiang, Shuxiang Li, Qiong Shen, Lei Chen

**Affiliations:** ^1^Department of Endocrinology, Suzhou Municipal Hospital, Nanjing Medical University, 26 Daoqian Road, Suzhou 215000, China; ^2^Department of Inspection, Suzhou Municipal Hospital, Nanjing Medical University, 26 Daoqian Road, Suzhou 215000, China

## Abstract

**Background:**

Autoimmune disorders are associated with gestational diabetes mellitus (GDM) in pregnant women who were positive for thyroid peroxidase antibody (TPOAb). Soluble CD40 ligand (sCD40L) and soluble interleukin-2 receptor (sCD25) are abnormally expressed in autoimmune diseases and are reliable markers of inflammation. The purpose of this study was to evaluate sCD40L and sCD25 in early pregnancy and investigate their correlation with GDM and TPOAb.

**Methods:**

A total of 126 pregnant women in the first trimester were enrolled for analysis: 93 were positive for TPOAb and 33 were negative for TPOAb. Demographical and clinical data in early pregnancy were collected. A total of 123 participants underwent a 75 g oral glucose tolerance test in the second trimester. Serum sCD40L and sCD25 levels were measured by ELISA.

**Results:**

The incidence of GDM was 24.4% in pregnant women with isolated TPOAb positivity in our study. Both sCD40L and sCD25 were positively correlated with TPOAb (*r* = 0.476, *P* < 0.001; *r* = 0.188, *P* < 0.05). sCD40L was highest in (*P* < 0.001) Ab-positive women with GDM group (*P* < 0.05). After adjusting for TPOAb, age, TSH, FT4, triglycerides, and low-density lipoprotein cholesterol, multivariate logistic regression analysis showed that sCD40L was an independent risk factor for GDM in pregnant women with TPOAb positivity (odds ratio = 3.235, 95% confidence interval 1.024–10.218, *P* < 0.05).

**Conclusions:**

About a quarter of pregnant women with isolated positive TPOAb might have GDM. sCD40L was an independent risk factor for GDM in women with isolated TPOAb positivity.

## 1. Introduction

Thyroid hormones are important determinants of metabolism and development for both mothers and fetuses [1]. Thyroid autoimmunity affects 8–14% of women of reproductive age and is characterized by the detection of thyroid peroxidative antibodies (TPOAb) or thyroglobulin antibody (TgAb) in circulation [2]. Thyroid dysfunction in autoimmune thyroid disease or isolated positive thyroid antibodies during pregnancy can result in adverse outcomes for the mother and fetus; such outcomes include recurrent spontaneous abortion, premature delivery, stillbirth, and fetal growth restriction [3].

Studies have suggested that gestational diabetes mellitus (GDM) occurs more often in pregnant women with thyroid disease [4, 5]. Subclinical hypothyroidism with TPOAb positivity is associated with GDM, but antibody negativity was not found to be related to GDM [6]. Isolated positivity for TPOAb was defined as euthyroid with TPOAb positivity in circulation. It was reported that the presence of TPOAb but not thyroglobulin antibodies (TgAb) was associated with a higher risk of GDM [7]. Few studies have concentrated on whether isolated TPOAb is associated with GDM. Huang et al. reported that isolated TPOAb positivity with euthyroidism in early pregnancy independently predicted GDM in a longitudinal observational study [8]. The higher the TPOAb titers, the higher the incidence of GDM. However, the underlying mechanism remains unknown.

The activation of T cells and their related cytokines contributes to the pathogenesis of autoimmune thyroiditis [9]. It was estimated that a dysregulated inflammatory response and isolated TPOAb positivity were involved in the pathophysiology of GDM [5, 9]. Costimulatory molecules are closely associated with T-cell activation. They function during the immune response and play critical roles in the production of thyroid antibodies [10]. CD40 ligand is mostly expressed on activated CD4+ T cells and interacts with CD40 on the B-cell surface. It plays an important role in the humoral response [11]. Soluble CD40 ligand (sCD40L) is the soluble form of the membrane-bound CD40 ligand that is released from activated platelets, activated CD4+ T cells, smooth muscle cells, and antigen-presenting cells [12]. In nonpregnant autoimmune thyroiditis patients, sCD40L was reported to be significantly higher than in patients without autoimmune thyroiditis, and it was positively correlated with TPOAb titers [13]. CD25 is the *α* subunit of interleukin-2 receptor (IL-2R) and is expressed on regulatory T (Treg) cells. It can affect the function of Treg cells, which are necessary to maintain maternal-fetal immune tolerance [14]. The soluble form of CD25 (sCD25) is produced by the proteolytic cleavage of the *α* subunit of IL-2R. The levels of IL-2R are stable in the steady state and are considered to be a marker of disease activity in many inflammatory diseases [15]. Thus, changes in costimulatory molecules in pregnant women with autoimmune thyroiditis could be of special interest.

Little is known about whether there is an interplay between TPOAb, GDM, and costimulatory molecules. This observational study assessed the role of sCD40L and sCD25 in pregnant women with TPOAb positivity. We compared the serum levels of sCD40L and sCD25 in pregnant women that were positive for TPOAb and those that were negative for TPOAb in the first trimester and investigated their relationship to GDM.

## 2. Participants and Methods

### 2.1. Participants

**Figure 1:**
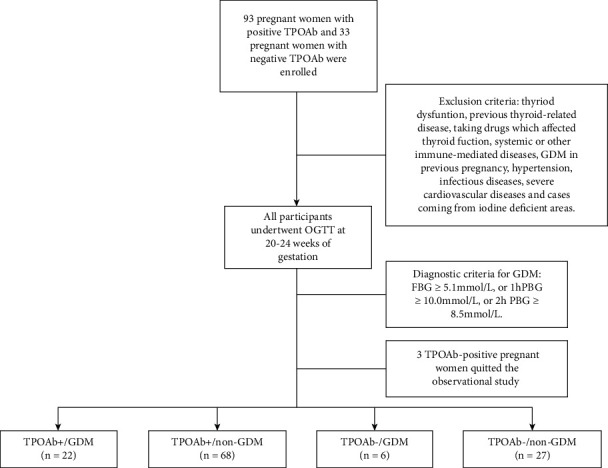
Flow diagram of the study process.

This single-center observational study was conducted in Suzhou Hospital Affiliated with Nanjing Medical University. Between June 2020 and December 2020, pregnant women in the first trimester who underwent a routine obstetric examination in our hospital were recruited for analysis. To eliminate the effect of thyroid hormones, all participants in our study were euthyroid which was defined as normal TSH and normal FT4 according to the routine thyroid function test in early pregnancy. All pregnant women received detailed medical history inquiries to collect personal information such as age, gestational weeks, prepregnancy weight, prepregnancy height, economic status of the family, and personal or family history of thyroid disease. Clinical data of glycosylated hemoglobin A1c (HbA1c), fasting plasma glucose (FPG), uric acid (UA), total cholesterol (TC), triglyceride (TG), high-density lipoprotein cholesterol (HDL-c), low-density lipoprotein cholesterol (LDL-c), c-reactive protein (CRP), white blood cell (WBC), red blood cell (RBC), hemoglobin (Hb), and platelet (Plt) was also collected. Exclusion criteria: thyroid dysfunction, previous thyroid-related diseases, taking drugs that affected thyroid function, systemic or other immune-mediated diseases, gestational diabetes mellitus in previous pregnancy, hypertension, infectious diseases, severe cardiovascular diseases, and cases coming from iodine-deficient areas. The flow diagram of our study is summarized in Figure 1.

The review board of the medical ethics committee in Suzhou Municipal Hospital approved the study protocol. Also, a written informed consent was provided to each participant and was signed before the survey.

### 2.2. Laboratory Measurement

Serum thyrotropin (TSH), free thyroxine (FT4), and TPOAb were measured in routine obstetric examination in weeks 10–13. Serum levels of TSH, FT4, and TPOAb were measured using automated chemiluminescent immunoassays (Architect i2000SR; Abbott Laboratories, Chicago, IL). The functional sensitivity of serum TSH was 0.0036 mIU/L. The intra-assay coefficients of variation (CV) of TSH, FT4, and TPOAb were 1.3∼6.3% and the inter-assay CV values were 2.0%∼6.6%. The laboratory reference ranges used in this study were provided by the manufacturer, as follows: TSH 0.35∼ 4.94 *μ*IU/mL, FT4 9.01∼19.04 pmol/L, and TPOAb < 12 IU/ml.

### 2.3. 75 g Oral Glucose Tolerance Test

75g oral glucose tolerance test (OGTT) was performed at 20–24 weeks gestation. Based on the diagnostic criteria recommended by the International Association of Diabetes and Pregnancy Study Groups (IADPSG) in 2010 [16], gestational diabetes mellitus (GDM) was diagnosed by fasting blood glucose (FBG) ≥ 5.1 mmol/L or 1 h postprandial blood glucose (1hPBG) ≥ 10 mmol/L or 2hPBG ≥ 8.5 mmol/L by OGTT. Normal glucose tolerance (NGT) was identified when FBG < 5.1 mmol/L and 1hPBG < 10 mmol/L and 2hPBG < 8.5 mmol/L.

### 2.4. sCD40L and sCD25 Measurement

Fasting blood samples were collected between 8 : 00 am and 10 : 00 am. The blood sample was centrifuged at 3000 rpm for 10 min. Serum was stored in sterilization EP tubes at −80°C and transferred to the central laboratory for centralized measurements of sCD40L and sCD25. Serum sCD40L and sCD25 concentrations were measured by ELISA kits (Xvguang Kexing Antibody Biotechnology Co., Ltd). Intra-assay and interassay variability coefficients for sCD40L and sCD25 were less than 5.00%. All samples were tested in the same batch to minimize interassay variability.

### 2.5. Statistical Analysis

All statistics were analyzed using SPSS Statistics v26.0 (SPSS, Inc.) and values of *P* < 0.05 were considered significant. The data were tested for normality using the Kolmogorov–Smirnov test. Data were presented as mean ± standard deviation for normally distributed variables or median (5% and 95% inter-quartiles) for nonnormally distributed variables. The value of TSH and TPOAb were log 10-normal-transformed to achieve normal distribution. We summarized demographic and laboratory characteristics as medians and interquartile ranges for continuous variables, or numbers and percentages for categorical variables. Statistical difference was evaluated using the Mann–Whitney *U* test for nonnormally distributed variables. Fisher's exact tests were used to test for the analysis of categorical variables.

Positive TPOAb was defined as a TPOAb titer higher than the reference upper limit. To study the association between sCD40L or sCD25 and TPOAb, we divided the study population into categories of TPOAb. According to TPOAb level, we divided the study population into TPOAb ≤ 12 IU/mL represented of the negative TPOAb group, 12 IU/mL < TPOAb ≤ 500 IU/mL represented of low titers of the positive TPOAb group, and TPOAb ≥500 IU/mL represented of high titers of the positive TgAb group.

To analyze the association of TPOAb, sCD40L, and sCD25 and the risk of GDM, we then divided the participants into 4 groups according to TPOAb positivity and OGTT results. Comparisons of clinical data between the four groups were performed by ANOVA test and Bonferroni correction was used for post hoc tests. Logistic regression analysis was used to explore the risk factors of GDM in positive TPOAb euthyroid participants.

## 3. Results

### 3.1. The Baseline and Demographical Characteristics of the Participants

A total of 126 pregnant women were finally analyzed in our study. 93 pregnant women were with positive TPOAb and 33 pregnant women were with negative TPOAb. All participants enrolled in our study were euthyroid. The mean TSH values were 1.83 ± 0.71 *µ*IU/L in the positive TPOAb group and 1.24 ± 0.87*µ*IU/L in the negative TPOAb group. TSH values were significantly higher in the positive TPOAb group than in the negative TPOAb group (*P* < 0.05). TG in the positive TPOAb group was significantly higher than that in the negative TPOAb group (1.59 ± 0.72 mmol/L *vs* 1.32 ± 0.37 mmol/L, *P* < 0.05). There were no significant differences in age, gestational weeks, gravidity, parity, prepregnancy BMI, HbA1c, FPG, UA, TC, HDL-c, LDL-c, CRP, WBC, RBC, Hb, Plt between the positive TPOAb group and the negative TPOAb group.

Three TPOAb-positive pregnant women quit the observational study. 123 participants underwent 75g OGTT in 24–26 gestational weeks. 22 (24.4%) participants were diagnosed of GDM in the positive TPOAb group and 6 GDM (18.18%) patients in the negative TPOAb group.

### 3.2. Serum sCD40L and sCD25 Levels Were Increased and Positively Correlated with TPOAb Titers

**Figure 2:**
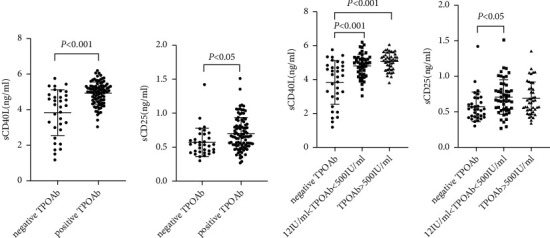
The comparison of sCD40L and sCD25 according to TPOAb positivity and TPOAb titers (a) showed the comparison of sCD40L in the negative TPOAb group and the positive TPOAb group; (b) showed the comparison of sCD25 in the negative TPOAb group and the positive TPOAb group; (c) showed the comparison of sCD40L according to TPOAb titers; (d) showed the comparison of sCD25 according to TPOAb titers. Comparisons of sCD40L and sCD25 levels between the three groups were performed by ANOVA test and Bonferroni correction was used for post hoc tests.

As shown in Table 1, sCD40L and sCD25 levels in the positive TPOAb group were significantly higher than that of controls (4.93 ± 0.62 ng/ml *vs* 3.83 ± 1.29 ng/ml, 0.70 ± 0.24 ng/ml *vs* 0.57 ± 0.21 ng/ml; *P* < 0.001, *P* < 0.05, Figures 2(a) and 2(b)). The subjects were further divided into 3 groups according to TPOAb titers, the TPOAb negative group (<12 IU/ml), low titers of the TPOAb group (12∼500 IU/ml), and high titers of the TPOAb group (>500 IU/ml). sCD40L was highest in high titers of the TPOAb group (3.83 ± 1.29 ng/ml *vs* 4.81 ± 0.69 ng/ml *vs* 5.06 ± 0.51 ng/ml, *P* < 0.05, Figure 2(c)). The levels of sCD25 in the low and high titer groups were higher than that in the negative TPOAb group (0.57 ± 0.21 ng/ml *vs* 0.71 ± 0.25 ng/ml *vs* 0.69 ± 0.23 ng/ml, *P* < 0.05, Figure 2(d)).

**Figure 3:**
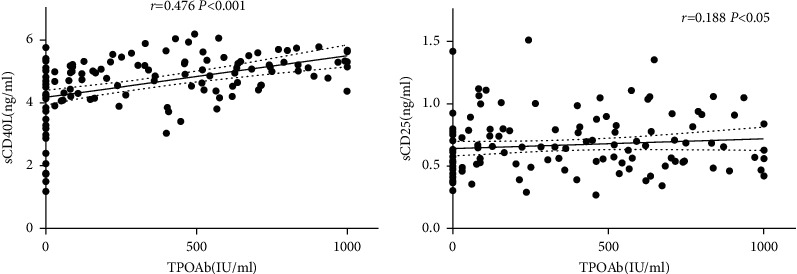
Correlation analysis between sCD40L, sCD25, and TPOAb titers.

Correlation analysis shows that sCD40L was positively correlated with the titers of TPOAb (*r* = 0.476; *P* < 0.001; Figure 3). TSH, FT4, TG, TC, HDL-c, LDL-c, and UA were not correlated with sCD40L. sCD25 is positively correlated with TPOAb (*r* = 0.188; *P* < 0.05; Figure 3) and UA (*r* = 0.213; *P* < 0.05). There was no correlation between TSH, FT4, TG, TC, HDL-c, LDL-c levels, and sCD25.

### 3.3. sCD40L Was Higher in GDM Patients with Positive TPOAb in the First Trimester

We found a higher incidence of GDM in positive TPOAb participants. To further analyze the relationship between TPOAb and GDM, participants were then divided into 4 groups: positive TPOAb with GDM, positive TPOAb without GDM, negative TPOAb with GDM, and negative TPOAb without GDM. In general, positive TPOAb with GDM group had higher TSH, lower FT4, and higher TG and LDL-c. TSH was highest in positive TPOAb without GDM group (1.90 ± 0.71 *µ*IU/L, *P* < 0.05). TSH was higher in positive TPOAb with GDM group than in negative TPOAb with GDM group (1.64 ± 0.73 µIU/L vs 1.32 ± 0.92 *µ*IU/L, *P* < 0.05). FT4 was lowest in the positive TPOAb group with GDM (12.60 ± 1.38 pmol/L, *P* < 0.05). TG and LDL-c were higher in the positive TPOAb with GDM group (1.92 ± 0.79 mmol/L, 2.82 ± 0.72 mmol/L, respectively; *P* < 0.05) In addition, sCD40L levels were the highest in positive TPOAb with GDM group compared to other 3 groups.

### 3.4. sCD40L Was an Independent Risk Factor for GDM in Pregnant Women with Positive TPOAb

**Figure 4:**
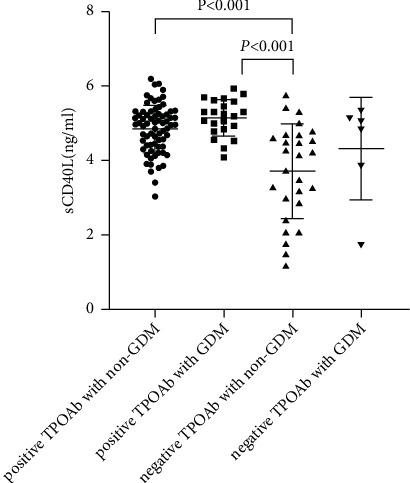
The comparison of sCD40L according to TPOAb positivity and the clinical fate of GDM. Comparison of sCD40L levels between the four groups was performed by ANOVA test and Bonferroni correction was used for post hoc tests.

As shown in Table 2 and Figure 4, sCD40L was significantly highest compared to other groups (*P* < 0.05). After adjusted for TPOAb, age, TSH, FT4, TG, and LDL-c, multivariate logistic regression analysis showed that sCD40L was an independent risk factor for GDM in pregnant women with positive TPOAb (odds ratio (OR) = 3.235, 95% confidential interval (CI) 1.024–10.218, *P* < 0.05, Table 3). In women with negative TPOAb, the odds ratio of sCD40L in GDM and non-GDM was 2.146 (95% CI 0.479–9.621, *P* = 0.319, Table 3).

## 4. Discussion

Our study investigated the relationships between TPOAb, sCD40L, and sCD25 levels in early pregnancy and the risk of GDM in pregnant women with or without TPOAb positivity. We found that sCD40L and sCD25 levels were significantly increased and positively correlated with TPOAb. In accordance with previous literature, pregnant women with TPOAb positivity had a higher incidence of GDM. After adjusting for the confounding factors of age, TSH, FT4, TG, and LDL-c, sCD40L levels were an independent predictor of GDM in early pregnancy.

Thyroid autoimmunity was recognized by the presence of antithyroid antibodies in circulation. And it was reported to have adverse effects on pregnancy and fetal development [1–3]. GDM is one of the most common complications in pregnancy [17]. It was documented that GDM occurred more often in pregnant women with thyroid disease. Subclinical hypothyroidism with TPOAb positivity was associated with GDM, while antibody negativity was not found to be related to GDM [6]. In terms of TPOAb and TgAb in GDM, it was found that the presence of TPOAb, not TgAb, was associated with GDM [7]. The rate of TPOAb positivity was higher in women with GDM than in women without GDM [7, 8, 18]. A meta-analysis of 11 studies found that there was no correlation between serum TSH levels and GDM. However, when TSH levels were combined with TPOAb positivity, the risk of GDM was increased [19]. Isolated TPOAb positivity with euthyroid in early pregnancy independently predicted GDM in a longitudinal observational study [7, 18], which was in accordance with our study. To eliminate the possible effect of thyroid dysfunction on GDM, we enrolled euthyroid pregnant women in our study. The incidence of GDM in the TPOAb-positive group was 24.4%, which was higher than that in the TPOAb-negative group (18.18%). The incidence of GDM was higher in the TPOAb-negative group in our study than in previous studies [17, 20]. Due to possible selection bias and the small sample size, no statistical significance was found in the incidence of GDM between the TPOAb-positive group and the TPOAb-negative group. Since all participants in our study were euthyroid, the association between TPOAb positivity and GDM might be a consequence of an autoimmune disorder.

To analyze the possible risk factors for TPOAb positivity in GDM during pregnancy, the participants were divided into 4 groups according to TPOAb positivity and GDM. We found that pregnant women with TPOAb positivity who were diagnosed with GDM had higher TSH and lower FT4 levels in early pregnancy. The difference in TPOAb positivity resulted in a difference in TSH and FT4. A higher TG and LDL-c were also found in our study in pregnant women with TPOAb positivity who were diagnosed with GDM. Due to increasing circulating placental hormones and increased maternal adiposity, progressive insulin resistance was found in GDM in early pregnancy and at its greatest in the second to third trimester [17, 21]. Insulin resistance could result in higher free fatty acids and hyperlipidemia in GDM. Thus, higher TG and LDL-c levels in pregnant women with TPOAb positivity who were diagnosed with GDM might be partially associated with the pre-existing insulin resistance in early pregnancy. Specifically, we found that sCD40L was highest in TPOAb-positive participants with GDM. And regression analysis showed that sCD40L was an independent risk factor for GDM after adjusting for age, TSH, FT4, LDL-c, and TG.

Costimulatory molecules play vital roles in initiating, maintaining, and exacerbating the immune response in autoimmune diseases [10]. Previous studies have found that the CD40/CD40 L system plays important roles in the regulation of the humoral response in autoimmune thyroiditis [11]. When T cells were activated, membrane CD40 L on CD4+ T cells increased and was then released into circulation. sCD40L levels were increased and correlated with autoantibodies and the severity of autoimmune diseases, such as rheumatoid arthritis, systemic lupus erythematosus, and multiple sclerosis [21–23]. In nonpregnant patients with thyroid autoimmunity, sCD40L levels were reported to be associated with TPOAb titers [13, 24, 25]. We found that sCD40L levels were increased and positively correlated with TPOAb titers in pregnant women and no correlation was found between sCD40L and either the TSH or FT4 concentration, which was in accordance with a previous study. sCD40L might be a good indicator of the severity of the inflammatory response in TPOAb-positive women. sCD40L was also a predictor of GDM in early pregnancy in our study which suggested an augment of inflammation and its related cytokines might be associated with the initiation of GDM in autoimmune thyroiditis. In a cross-sectional and longitudinal study of women with and without GDM, an elevation of monocyte-chemoattractant-protein-1(MCP-1) not sCD40L was found to be associated with GDM [26]. The difference in TPOAb positivity in each study might also be the explanation for the difference.

Increased sCD25 levels in pregnant women positive for TPOAb were also found in our study. However, we did not find any correlation between sCD25 and GDM. sCD25 is an important laboratory parameter that is used to detect T-cell activity and immunomodulatory function, which is elevated in many autoimmune diseases [27]. An in vitro study showed that the blockade of the sCD25-mediated IL-2 signaling pathway enhances T-cell responses to the Th17 phenotype and promotes the development of autoimmune diseases [28-29]. A positive correlation with UA levels was observed. Uric acid directly promotes human T-cell activation, as reported by Ryan Webb [30]. Due to the complex change in immune tolerance in pregnancy, the causal relationship between elevated sCD25 levels and the pathogenesis of autoimmune diseases needs to be further explored.

The strengths of our study were as follows. First, we adopted the pregnancy-specific reference range of thyroid hormones in our study. Second, all participants enrolled and analyzed in our study were euthyroid. The possible effects of thyroid dysfunction were eliminated. Third, we found that costimulatory molecules were a good indicator of the severity of autoimmunity and had a predictive role for GDM in early pregnancy for the first time.

This study has some limitations. First, the cross-sectional survey and the small number of subjects are the main limitations of this study. Therefore, it is impossible to conclude whether higher levels of sCD40L and sCD25 are directly involved in the pathogenesis of TPOAb positivity in GDM or just a result of the immune-mediated process. Second, we did not have longitudinal data on thyroid hormones before and after pregnancy. Third, we did not have available data on the iodine nutritional status of the participants. However, according to the latest epidemic study of the iodine nutritional status in 31 cities in China, China is an iodine-sufficient country[31] [30].

## 5. Conclusions

Pregnant women who are positive for TPOAb and euthyroid presented with higher TG, higher TSH, and lower FT4 in early pregnancy, and they had a higher risk of developing GDM. sCD40L and sCD25 levels are increased and positively correlated with TPOAb titers in pregnant women. sCD40L was an independent risk factor for GDM in pregnant women with TPOAb positivity.

## Figures and Tables

**Table 1:** Baseline and demographical characteristics and laboratory parameters of TPOAb-positive pregnancies and TPOAb-negative controls.

	TPOAb-positive	TPOAb-negative	*P* Value
Age (y)	30.19 ± 3.99	29 ± 2.94	0.118
Gestational weeks	13.18 ± 2.16	12.98 ± 1.66	0.633
Prepregnancy BMI (kg/m^2^)	22.15 ± 2.88	21.80 ± 2.70	0.543
Gravida	2 (1–6)	2 (1–4)	0.124
Parity	0 (0–2)	0 (0–1)	0.812
Medically assisted reproduction	6 (6.45%)	4 (12.12%)	0.301
TSH (µIU/L)	**1.83** **±** **0.71**	**1.24** **±** **0.87**	<0.001
FT4 (pmol/L)	12.76 ± 1.31	13.59 ± 2.41	0.067
HbA1c (%)	5.18 ± 0.29	5.16 ± 0.30	0.829
FPG (mmol/L)	4.59 ± 0.39	4.68 ± 0.36	0.244
UA (mmol/L)	209.46 ± 51.95	224.68 ± 42.80	0.134
TG (mmol/L)	**1.59** **±** **0.72**	**1.32** **±** **0.37**	<0.05
TC (mmol/L)	4.92 ± 0.81	4.82 ± 0.84	0.513
HDL-c (mmol/L)	1.83 ± 0.36	1.84 ± 0.33	0.923
LDL-c (mmol/L)	2.56 ± 0.68	2.54 ± 0.75	0.881
CRP (mmol/L)	2.76 (1.33–4.63)	1.35 (0.97–3.78)	0.091
WBC (×10^9^/L)	8.61 ± 1.86	8.57 ± 1.34	0.906
RBC (×10^12^/L)	4.21 ± 0.41	4.16 ± 0.27	0.540
Hb (g/L)	127.27 ± 9.79	127.30 ± 9.14	0.986
Plt (×10^9^/L)	229.72 ± 54.17	231.91 ± 55.07	0.843
GDM (N, %)	22, 24.4%	6, 18.18%	0.628
sCD40L(ng/mL)	4.93 ± 0.62	3.83 ± 1.29	<0.001
sCD25(ng/mL	0.70 ± 0.24	0.57 ± 0.21	<0.05

*∗P* < 0.05 is statistically significant. BMI, body mass index; TPOAb, thyroid peroxidase antibody; TSH, thyroid-stimulating hormone; FT4, free thyroxine; HbA1c, glycosylated hemoglobin A1c; FPG, fasting plasma glucose; UA, uric acid; TG, triglyceride; TC, total cholesterol; HDL-c, high-density lipoprotein cholesterol; LDL-c, low-density lipoprotein cholesterol; CRP, C-reactive protein; WBC, white blood cell; RBC, red blood cell; Hb, hemoglobin; Plt, platelet; GDM, gestational diabetes mellitus; sCD40L, soluble CD40 ligand; sCD25, soluble CD25.

**Table 2:** The comparison of baseline and laboratory parameters of participants with or without GDM in early pregnancy.

Variables	TPOAb-positive		TPOAb-negative		*P* Value
	Non-GDM	GDM	Non-GDM	GDM	
Age (y)	29.69 ± 3.69	32.14 ± 4.4^ac^	29.3 ± 2.89	27.67 ± 3.01	<0.05
Gestational weeks	13.17 ± 2.27	13.30 ± 1.95	12.91 ± 1.72	13.31 ± 1.45	0.916
Prepregnancy BMI(kg/m2)	21.81 ± 2.70	23.49 ± 3.05	21.98 ± 2.64	20.97 ± 3.07	0.068
Gravida	2 (1–3)	2 (1–3)	2 (1–2)	2 (1–3)	0.544
Parity	0 (0-1)	0 (0–1)	0 (0–1)	0 (0–1)	0.783
TSH(µIU/mL)	1.90 ± 0.71^ab^	1.64 ± 0.73	1.32 ± 0.92	0.90 ± 0.55	0.001
FT4(pmol/L)	12.76 ± 1.27	12.60 ± 1.38	13.76 ± 2.53	12.85 ± 1.82	<0.05
UA(µmol/L)	207.73 ± 54.41	215.64 ± 47.56	226.00 ± 42.63	218.75 ± 47.17	0.454
TG(mmol/L)	1.51 ± 0.68	1.92 ± 0.79^a^	1.33 ± 0.35	1.25 ± 0.47	<0.05
TC(mmol/L)	4.83 ± 0.79	5.26 ± 0.82	4.90 ± 0.87	4.42 ± 0.60	0.082
HDL-c(mmol/L)	1.81 ± 0.36	1.91 ± 0.37	1.82 ± 0.35	1.95 ± 0.23	0.588
LDL-c(mmol/L)	2.49 ± 0.65	2.82 ± 0.72	2.66 ± 0.75	2.01 ± 0.53	<0.05
CRP(mg/L)	2.46 (1.15–3.95)	3.85 (2.00–6.64)	1.35 (1.04–3.95)	1.74 (0.68–3.38)	0.134
WBC(x10^9^/L)	8.43 ± 1.85	9.24 ± 1.90	8.49 ± 1.28	8.96 ± 1.66	0.269
RBC(x10^12^/L)	4.18 ± 0.42	4.33 ± 0.37	4.16 ± 0.26	4.17 ± 0.33	0.365
Hb(g/L)	126.65 ± 10.01	130.05 ± 9.17	128.33 ± 8.70	122.67 ± 10.44	0.290
Plt(x10^9^/L)	228.76 ± 54.74	232.55 ± 56.28	232.81 ± 56.99	227.83 ± 49.95	0.984
sCD40L(ng/mL)	4.84 ± 0.63^a^	5.15 ± 0.49^a^	3.72 ± 1.27	4.32 ± 1.37	<0.001
sCD25(ng/mL)	0.69 ± 0.24	0.70 ± 0.20	0.56 ± 0.15	0.61 ± 0.40	0.073

Adjustment for multiple comparisons: Bonferroni correction.(a) compared with negative-TPOAb women without GDM, *P* < 0.05.(b) compared with negative-TPOAb women with GDM, *P* < 0.05.(c) compared with positive-TPOAb women without GDM, *P* < 0.05. BMI, body mass index; TPOAb, thyroid peroxidase antibody; TSH, thyroid-stimulating hormone; FT4, free thyroxine; UA, uric acid; TG, triglyceride; TC, total cholesterol; HDL-c, high-density lipoprotein cholesterol; LDL-c, low-density lipoprotein cholesterol; sCD40L, soluble CD40 ligand; sCD25, soluble CD25.

**Table 3:** Multivariable logistic regression analysis of risk factors for GDM in pregnant women with positive TPOAb.

Predictor	*β*	SE	Wald X^2^	*P* Value	*OR*	95% *CI*
TPOAb	0.000	0.001	0.004	0.949	1.000	0.998–1.002
sCD40L	1.174	0.587	4.005	0.045	3.235	1.024–10.218
TSH	−0.435	0.456	0.910	0.340	0.647	0.265–1.582
FT4	−0.008	0.243	0.001	0.975	0.992	0.617–1.597
TG	0.554	0.413	1.794	0.180	1.740	0.774–3.911
LDL-c	0.388	0.448	0.753	0.385	1.475	0.613–3.546
Age	0.151	0.068	4.930	0.026	1.164	1.018–1.330

TPOAb, thyroid peroxidase antibody; sCD40L, soluble CD40 ligand; TSH, thyroid-stimulating hormone; FT4, free thyroxine; TG, triglyceride; LDL-c, low-density lipoprotein cholesterol.

## Data Availability

The clinical data of the population used to support the findings of this study are available from the corresponding author upon request.
